# Precision Therapy for a Chinese Family With Maturity-Onset Diabetes of the Young

**DOI:** 10.3389/fendo.2021.700342

**Published:** 2021-08-05

**Authors:** Juyi Li, Meng Shu, Xiufang Wang, Aiping Deng, Chong Wen, Juanjuan Wang, Si Jin, Hongmei Zhang

**Affiliations:** ^1^Department of Pharmacy, The Central Hospital of Wuhan, Tongji Medical College, Huazhong University of Science and Technology, Wuhan, China; ^2^Department of Endocrinology, Institute of Geriatric Medicine, Liyuan Hospital, Tongji Medical College, Huazhong University of Science and Technology, Wuhan, China; ^3^Department of Pain, The Central Hospital of Wuhan, Tongji Medical College, Huazhong University of Science and Technology, Wuhan, China; ^4^Department of Traditional Chinese Medicine, The Central Hospital of Wuhan, Tongji Medical College, Huazhong University of Science and Technology, Wuhan, China; ^5^Department of Endocrinology, The Central Hospital of Wuhan, Tongji Medical College, Huazhong University of Science and Technology, Wuhan, China

**Keywords:** glucokinase, monogenic diabetes, mutation, MODY2, pedigree

## Abstract

**Objective:**

To determine the pathogenic gene and explore the clinical characteristics of maturity-onset diabetes of the young type 2 (MODY2) pedigree caused by a mutation in the glucokinase (*GCK*) gene.

**Methods:**

Using whole-exome sequencing (WES), the pathogenic gene was detected in the proband—a 20-year-old young man who was accidentally found with hyperglycemia, no ketosis tendency, and a family history of diabetes. The family members of the proband were examined. In addition, relevant clinical data were obtained and genomic DNA from peripheral blood was obtained. Pathologic variants of the candidate were verified by Sanger sequencing technology, and cosegregation tests were conducted among other family members and non-related healthy controls. After adjusting the treatment plan based on the results of genetic testing, changes in biochemical parameters, such as blood glucose levels and HAblc levels were determined.

**Results:**

In the *GCK* gene (NM_000162) in exon 9, a heterozygous missense mutation c.1160C > T (p.Ala387Val) was found in the proband, his father, uncle, and grandmother. Thus mutation, which was found to co-segregate with diabetes, was the first discovery of such a mutation in the Asian population. After stopping hypoglycemic drug treatment, good glycemic control was achieved with diet and exercise therapy.

**Conclusion:**

*GCK* gene mutation c.1160C > T (p.Ala387Val) is the pathogenic gene in the *GCK-MODY* pedigree. Formulating an optimized and personalized treatment strategy can reduce unnecessary excessive medical treatment and adverse drug reactions, and maintain a good HbA1c compliance rate

## Introduction

Maturity-onset diabetes of the young (MODY) is a genetically heterogeneous type of monogenic diabetes mellitus that is characterized by autosomal dominant inheritance, an early age at onset, and pancreatic β-cell dysfunction (1). MODY is a diabetes subtype that is distinct from type 1 diabetes (T1D) and type 2 diabetes (T2D) (2). Identification of the MODY subtype is critical due to important therapeutic implications (3). It shows a different clinical heterogeneity due to its different mutation genes, therefore, disease judgment, prognosis, and treatment are also different (4). So far, 14 MODY-related genes have been discovered, including *KCNJ11*, *ABCC8*, *INS*, *GCK*, *IPF1*, *PTF1A*, *GLIS3*, *FOX3*, *EIF2AK3*, *GLUT2*, *HNF1A*, *HNF1B*, *HNF4A*, and *PAX4* (5–9). Among these genes, heterozygous mutations in *HNF1A*, *HNF4A*, and *GCK* have been identified as the root cause of more than 90% of MODY cases (10). Moreover, mutations in *GCK*, *HNF1A*, and *HNF4A HNF1B* are the most common causes of MODY, accounting for 32%, 52%, 10% and 6% of all infected patients in the UK, respectively (3). The frequency of this causality, however, may differ between the Asian and Caucasian population. A genetic screening study among the Chinese population included 74 clinically highly suspected MODY patients from 59 unrelated families with a detection rate in *HNF1A* and *HNF4A* of 13.6% and 1.7%, respectively (11). In another MODY genetic screening study of 76 unrelated families, GCK, *HNF1A* and *HNF4A* accounted for 18.42%, 15.79% and 2.63% of all MODY cases, respectively (12). In the Chinese population, few cases of MODY have been identified, and investigation of large sample population is lacking. These data suggest that there may be unidentified genetic variants that play a role in MODY cases in the Asian population. Early identification and accurate classification of MODY cases is an important factor that is critical to guide precise treatment and determine patient prognosis.

The clinical manifestations of MODY depend on the molecular genetic basis. Patients with MODY resulting from mutations in genes that encode transcription factors present with more overt diabetes, often with a striking family history and frequent diabetic complications (13, 14). In contrast, patients with mutations in the *GCK* gene present with mild, stable hyperglycemia from birth, are mostly treated by diet alone and rarely suffer from diabetic complications (15, 16). In general, patients with *GCK-MODY* do not need pharmacological therapy (17). During illness or routine screening, genetic testing of family members can prevent the anxiety that is associated with hyperglycemia. Therefore, a correct genetically-based diagnosis of MODY is crucial to confirm the diagnosis and establish a treatment plan. Incorrect classification of MODY subtypes may lead to unnecessary treatment and increase personal healthcare expenditures.

Next generation sequencing is characterized by high throughput and low cost, which is conducive to clear research objectives and accuracy as well as for improving timeliness, to obtain required and high-quality results from the limited DNA information (18, 19). Application of this technique in the diagnosis of MODY diabetes improves the accuracy of clinical diagnosis.

Whole exome sequencing (WES) can improve molecular diagnostics of monogenic diabetes, thus genetic testing has important implications for MODY (20). In our study, WES was used to conduct genetic and functional studies on a family of suspected monogenic diabetes patients in China to identify disease-causing genes in a family and to explore the clinical characteristics of MODY patients caused by gene mutations. This may provide guidance for precise treatment of MODY and targeted individualized preventive measures for all carriers bearing a genetic mutation.

## Materials and Methods

### Participants

All subjects completed questionnaires regarding medical and family history, and information was supplemented by information from medical records. Diagnosis and the classification of diabetes complied with the latest guidelines presented by the American Diabetes Association (ADA) (21). This study was approved by the Ethics Committee of Wuhan Central Hospital, and participants signed informed consent for participation in the study.

### DNA Extraction and Whole Exome Sequencing

Clinical data were obtained from all patients suspected of MODY, and 5ml of fasting blood was collected using EDTA for anticoagulation purposes. According to the manufacturer’s instructions, a DNA Extraction Kit (Tiangen Biotech, Beijing, China) was used to isolate genomic DNA of peripheral blood obtained from the MODY family. For high-throughput sequencing, the SureSelect Human All Exon V5 kit (Agilent technologies Inc, USA) was used for sample preparation and pretreatment, and samples underwent WES using the Illumina HiSeq 2500 sequencing platform.

### Molecular Genetic Analyses

The sequence was aligned with the human genome reference sequence (UCSC hg19, GRCh37), compared, and the SNPs (Single Nucleotide Polymorphism) and InDels (Insertion-Deletion) variants were obtained after alignment with publicly available databases (1000 Genomes, ExAC (Exome Aggregation Consortium), dbSNP, ESP (NHLBI Exome Sequencing Project), and gnomAD (Genome Aggregation Database)). For mutations that were not detected in the database, further analysis of the impact that mutation on changes in the genetic code is required to distinguish between synonymous mutations and missense mutations. Next, several silico prediction tools (SIFT, PolyPhen2, Mutation Taster, Mutation Assessor, FATHMM, GERP-plus, PhyloP100, PhastCons100) were used to predict the influence of identified nonsynonymous mutations on protein structure and function.

### Sanger Sequencing

It is known that high-throughput sequencing has a certain rate of mismeasurement. Therefore, Sanger sequencing should be performed in the proband and his family to verify variants identified by next generation sequencing. Polymerase chain reaction (PCR) amplification of target gene fragments: Primer design: Based on the location of the mutation in the target gene, primers were designed at the genome-wide level. Primer synthesis: completed by Beijing Kinco Biotechnology Co., Ltd. Target gene amplification: the target gene was amplified using Golden Mix (green) Golden Star T6 Super PCR Mix (1.1×) from Beijing Kinco Biotechnology Co., Ltd. (Beijing, China). Subsequently, the amplified product was subjected to agarose gel electrophoresis, and the target band as cut out of the gel and recovered using a DNA gel imager. The PCR product fragments were sequenced as follows: After the PCR product was tapped, recovered, and purified, the product was sequenced using an ABI3730XL Automatic Sequencer (Applied Biosystems, Foster City, CA). The sequencing results were compared and analyzed with genome sequences that were downloaded from the Genebank database (using AutoAssembler 2.0 software).

### Structure Modeling

The Swiss-Prot repository (http://web.expasy.org/docs/swiss-prot_guideline.html) was used to simulate the three-dimensional (3D) structure of the protein sequence of the GCK gene bearing a missense mutation.

### Medication Plan Adjustment and Follow Up the Effect of Drug Treatment

Based on the genetic testing results and related literature reports, the patient’s medication plan was adjusted in a timely manner. Glycemic control was assessed *via* monitoring blood glucose levels of each subject (at least one blood glucose measurement per day) and by rechecking HbA1c levels.

## Results

### Characteristics of Study Participants

**Figure 1:**
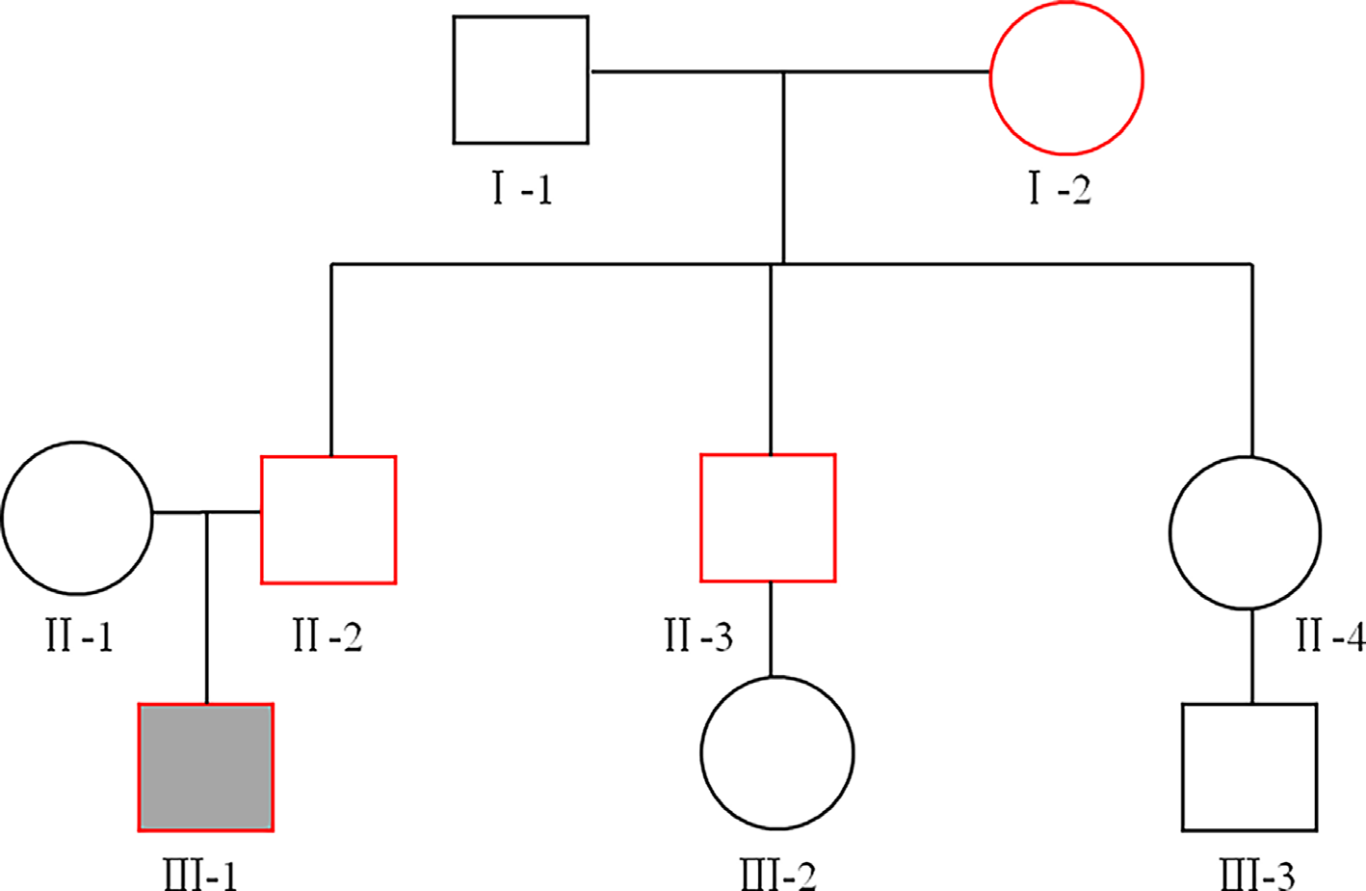
The family pedigree. Squares represent males, circles represent females. The proband is represented by a solid black box, and the red line indicates patients with diabetes. The proband (III-1), his father (II-2), his uncle (II-3), and his grandmother (I-2) were diagnosed with type 2 diabetes (T2D) at the age of 18, 42, 43, and 60, respectively.

Baseline characteristics of the study subjects are displayed in **Table 1**. The pedigree of this family is displayed in **Figure 1**. Physical and laboratory analyses showed that all subjects were negative for conventionally-assessed islet autoantibodies and urine sugar. Oral hypoglycemic drugs are ineffective for diabetic patients, and patients with diabetes show impaired beta-cell islet function. In this family, patients with diabetes were previously diagnosed as T2D. In addition, the proband’s grandmother (I-2) suffered from cataracts and previous suffered a stroke, but there were no sequelae. The proband’s father (II-2) had high blood pressure.

**Table 1:** Physical and laboratory examination.

	Ⅰ-2	Ⅱ-1	Ⅱ-2	Ⅱ-4	Ⅲ-1
Age, y	82	52	50	52	20
Gender	M	M	F	M	F
BMI (kg/m^2^)	19.1	23.4	23.9	25.3	19.6
FPG (3.9-6.1, mmol/l)	6.70	4.53	6.3	5.0	7.0
FCP (1.1-3.3, ng/ml)	--	--	--	--	2.0
HbA1c (4-6, %)	6.5	5.5	6.7	5.7	6.4

F, female; M, male; BMI, body mass index; FPG, fasting plasma glucose; FCP, Fasting C-peptide; HbA1c, glycosylated hemoglobin.

### Variant Detection

Exome Capture Statistics are shown in **Table 2**. In brief, a total of 130076 mutations were identified by WES, including 118031 SNPs and 12045 InDels. Among them, the numbers of non-synonymous SNVs (Single nucleotide variant), synonymous SNVs, frameshift deletions, frameshift insertions, non-frameshift deletions, and non-frameshift insertions were 56218, 58449, 2708, 2091, 3165, and 2389, respectively.

**Table 2:** Details of whole exome sequencing (WES).

Exome Capture Statistics	Proband
Target Region (bp)	60,456,963
Clean Reads	135,777,598
Clean Bases	13,577,759,800
Mapped Reads	135,549,849
Mapped Bases	13,533,054,631
Mapping Rate (%)	99.83
Reads Mapped to Target Region	66,111,905
Capture specificity (%)	48.77
Duplication Rate (%)	28.73
Uniq Rate(%)	98.05
Bases Mapped to Target Region	5,390,495,753
Mean Depth of Target Region	89.16
Coverage of Target Region (%)	96.14
Fraction of Target Covered >=4X	93.13
Fraction of Target Covered >=10X	89.06
Fraction of Target Covered >=20X	82.2
Fraction of Target Covered >=30X	74.55
Fraction of Target Covered >=50X	58.69
Bases Mapped to Flanking Region	2,434,278,684
Mean Depth of Flanking Region	51.83
Coverage of Flanking Region (%)	88.42
Fraction of Flanking Covered >=4X	80.14
Gender	Male

### Genetic and Bioinformatics Analyses

**Figure 2:**
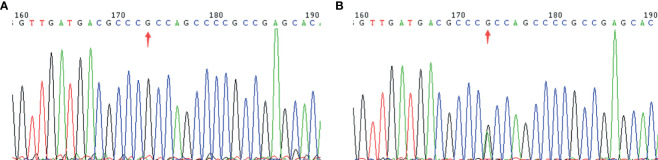
A mutation in the *GCK* gene causes MODY2. **(A)**: *GCK* wild type; **(B)**: *GCK* heterozygote.

To identify key biological functions in the family of the proband, variants were filtered according to their genome site location, allele frequency, and functional consequences, excluding intronic and nonsense variants. Mutation sites are presented in **Table 3**. Functional consequences of the coding variant were estimated by several prediction programs including SIFT, PolyPhen2, Mutation Taster, Mutation Assessor, FATHMM, GERP-plus, PhyloP100, and PhastCons100 (**Table 4**), and the results suggest that the alteration predicted was probably pathogenic, had a deleterious effect on protein performance, and was closely related to diabetes. Sequencing in other relatives of this family and 200 non-related healthy controls revealed that affected relatives (I-2, II-2, II-3) were heterozygous for the same mutation, which was not found in non-related healthy controls (**Tables 5**, **6**, and **Figure 2**). Taken together, the data demonstrate that only heterozygous missense mutations with predicted destructive effects were found in diabetic patients, thereby indicating that the variant in the *GCK* gene co-segregated with diabetes. The mutation was located on chromosome 7, position 44185189, c.1160C>T, NM_000162, p.Ala387Val, namely rs193921338. The p.Ala387Val mutation was analyzed for evolutionary conservation, which showed that the mutation was located in a highly conserved region among multiple animal species (**Table 7**). In the ExAC database (http://exac.broadinstitute.org/), the variant was categorized as a rare change with a mutation frequency of 0.00001145. In addition, in this database, the mutation was classified as likely pathogenic. Therefore, based on the findings described above and the clinical condition of this family, this rare missense mutation was considered a disease-causing mutation.

**Table 3:** Information of candidate pathogenic gene loci.

CHR	POS	ID	REF	ALT	GENE	HGVSc	HGVSp
7	44185189	rs193921338	G	A	*GCK*	c.1160C > T	p. Ala387Val

CHR, Chromosome; POS, position; ID, identification; REF, Reference; ALT, alternative; HGVSc, human genome variation society cDNA; HGVSp, human genome variation society protein; G, guanine; A, adenine.

**Table 4:** Pathogenicity results of candidate gene mutation sites predicted by bioinformatics analysis.

GENE	HGVSc	SIFT	PolyPhen2_HDIV	PolyPhen2_HVAR	MutationTaster	MutationAssessor	FATHMM	GERP_plus	PhyloP	PhastCons
*GCK*	c.1160C > T	0.107	1	0.991	1	1.945	-4.67	5.46	9.901	1

HGVSc, human genome variation society cDNA; SIFT, Deleterious(<0.05); PolyPhen2_HDIV, Probably damaging (>=0.957), possibly damaging (0.453<=pp2_hdiv<=0.956); benign (<=0.452); PolyPhen2_HVAR, Probably damaging (>=0.909), possibly damaging (0.447<=pp2_hdiv<=0.909); benign (<=0.446); MutationTaster, Deleterious(>0.5); MutationAssessor, Deleterious(>1.938); FATHMM, Deleterious(<-1.5); GERP_plus, Deleterious(>3); PhyloP, Deleterious(>2.5); PhastCons, Deleterious(>0.6).

**Table 5:** Sanger sequencing results of candidate gene loci in family members of the proband.

GENE	HGVSc	HGVSp	Member of family	ALT	REF
*GCK*	c.1160C > T	p.Ala387Val	I-2	T	C
			II-1	C	C
			II-2	T	C
			II-4	C	C
			III-1	T	C

HGVSc, human genome variation society cDNA; HGVSp, human genome variation society protein; ALT, alternative; REF, Reference; C, cytosine; T, thymine.

**Table 6:** Sanger sequencing results of the c.1160C > T mutation in the *GCK* gene in 200 non-related healthy controls.

Subjects	Number of people carrying the mutation
non-related healthy controls (*n*=200)	0

**Table 7:** Evolutionary conservation analysis of the p.Ala387Val mutation in the *GCK* gene.

Protein Acc.	Gene	Organism	Amino acid sequences		
NP_000153.1	*GCK*	H.sapiens	356	GLRPSTTDCDIVRRACESVSTRAAHMCSAGLAGVINRMRESRSEDVMRIT	405
XP_001143302.1	*GCK*	P.troglodytes	355	GLRPSTTDCDIVRRACESVSTRAAHMCSAGLAGVINRMRESRSEDVMRIT	404
XP_001093035.2	*GCK*	M.mulatta	356	GLRPSATDCDIVRRACESVSTRAAHMCSAGLAGVINRMRESRSEDVMRIT	405
NP_001095772.1	*GCK*	B.taurus	356	GLRPSATDCDIVRRACESVSTRAAHMCAAGLAGVINRMRESRSEDVMRIT	405
NP_034422.2	*Gck*	M.musculus	356	GLRPSVADCDIVRRACESVSTRAAHMCSAGLAGVINRMRESRSEDVMRIT	405
NP_036697.1	*Gck*	R.norvegicus	356	GLRPSVTDCDIVRRACESVSTRAAHMCSAGLAGVINRMRESRSEDVMRIT	405
XP_427930.4	*GCK*	G.gallus	449	GLLPSGSDCDIVRMVCESVSTRAAQMCSAGLAGVINRMRESRSQETLKIT	498
NP_001038850.2	gck	D.rerio	366	GI LPSELDCDIVRLACESVSTRAAHLCGAGLAGVINLMRERRCQEELKIT	415
NP_001096321.1	gck	X.tropicalis	349	GVQATIGDCHAVRLACESVSTRAAVMCSSGLAAILNRMHQSRRGELSRIT	398

A means alanine(Ala), in many species, the 387th amino acid of the GCK gene is alanine, indicating that the p.Ala387Val mutation is located in a highly conserved region.

### Prediction of Protein Structure

**Figure 3:**
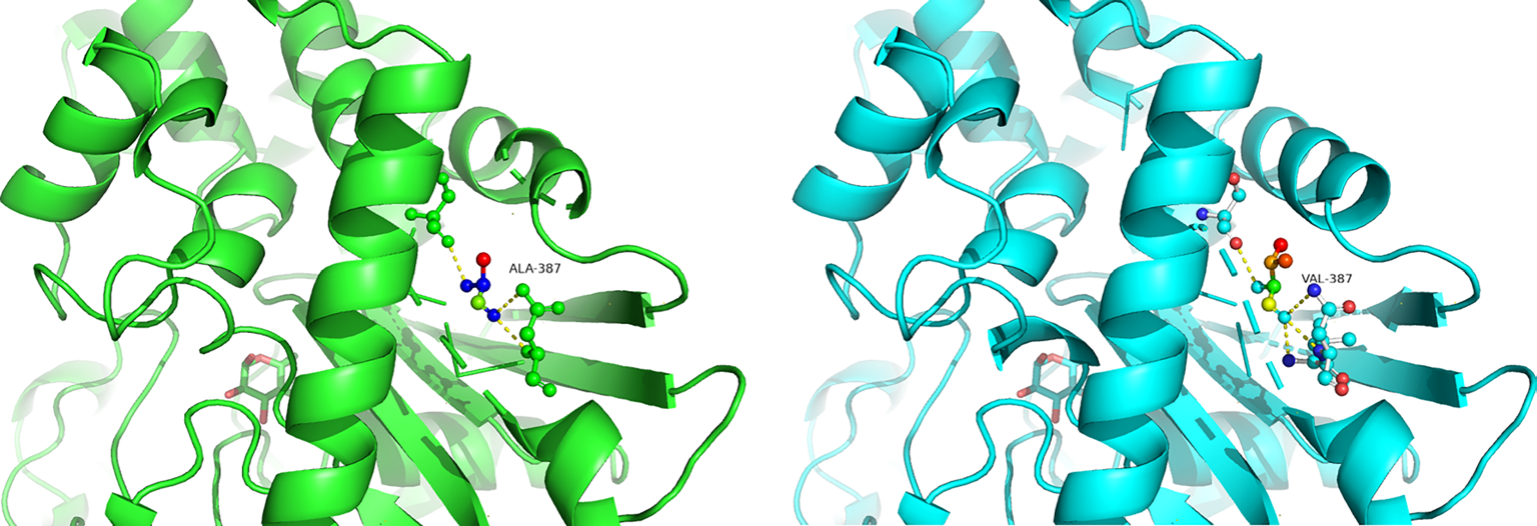
Predicted computational tertiary structure. The wild genotype is presented on the left; the mutant genotype is presented on the right.

Homology models of the tertiary protein structure of wild-type and p.Ala387Val GCK proteins were predicted by Swiss-Prot (**Figure 3**), and the mutated protein structure was compared with the normal (non-mutated) protein structure. A change in amino acids results from a nonsynonymous substitution. A close-up of amino acid change analysis shows that the number of chemical bonds and hydrophilicity of amino acid side chains have changed, which may cause structural disorder and change protein function.

### Clinical Follow-Up After Drug Withdrawal

To validate the type of diabetes caused by the mutation in the *GCK* gene, for which no additional hypoglycemic drugs are needed, a follow-up observation was performed after drug withdrawal (**Table 8**). The results of blood glucose monitoring showed that after discontinuation of an oral hypoglycemic drug (metformin), the proband’s blood glucose level was still maintained at a stable level and there was no need for excessive drug therapy to control his blood glucose level.

**Table 8:** Fluctuations of self-monitoring blood glucose (SMBG) and HbA1c during follow-up analysis.

Date	FPG (3.9~6.1, mmol/L)	PPG (4.4~7.8, mmol/L)	HbA1c (4~6%)
July 23, 2020~August 29, 2020	5.3~5.7	5.7~9.9	6.1

## Discussion

MODY is a type of monogenic diabetes that is inherited in an autosomal dominant pattern with different prevalence rates in different ethnic groups (22, 23). In this study, we used WES to study the sequence variations of all genes in a suspected MODY family of early-onset diabetes. In this family, there were 5 cases of abnormal glucose metabolism, among which the proband was diagnosed with diabetes when he was younger than 25 years of age. Three generations of his immediate family members were diagnosed with diabetes, which conforms to the characteristics of autosomal dominant inheritance and meets the diagnostic criteria for MODY (24). *GCK-MODY* is caused by a mutation in the *GCK* gene, which leads to decreased activity of this enzyme, and is one of the most common characteristics of MODY (25). In previous studies, it has been shown that the prevalence of MODY caused by the mutation in the *GCK* gene in the Chinese population is 0.21%, and the prevalence of MODY in Chinese diabetic population is 1.3% (26).

Patients with *GCK-MODY* present with a mild clinical phenotype, which does not lead to serious complications of diabetes (27–29). In general, blood glucose levels can be controlled by adjustments of diet and exercise. Insulin or oral hypoglycemic drugs are not effective in lowering the blood glucose levels or HbA1c levels in *GCK-MODY* patients, so there is no need to over-treat the patient (other than during pregnancy) (30). In the general population, in patients with *GCK-MODY* who are not pregnant, ensuring a lifestyle modification involving diet and exercise is often the only necessary intervention (31, 32). This can significantly help improve the patient’s quality of life (33). In our study, we found that adherence to a healthy diet and exercise can help patients with *GCK-MODY* achieve good control of their blood glucose level.

So far, 620 types of mutations have been identified in the *GCK* gene in a total of 1441 families (34, 35), and there are still many gene variants to be discovered. This study was the first to report a *GCK-MODY* family in the Chinese population bearing by a heterozygous missense mutation in the *GCK* gene c.1160C > T (p.Ala387Val). Data analysis showed that the mutation involved a rare change with a mutation frequency of 0.00001145 of which the variant was predicted to be likely pathogenic. Recent evidence has suggested that the coding region in which the mutation is located is a highly conserved region in many species. The mutation can cause changes in protein structure and affect protein stability, thereby affecting functional changes. Therefore, we have reason to believe that the *GCK* gene is the disease-causing gene in this diabetes family, and the pathogenic mutation site is rs193921338, which was first discovered in the Chinese population. For *GCK-MODY*, correct molecular genetic diagnosis is critical (36). Young patients, especially children, suffer from diabetes, and there is no typical clinical evidence of T1DM or T2DM. Therefore, *GCK-MODY* should be highly suspected, and further investigation and screening of the *GCK* gene mutation should be conducted in the future (37). These findings will help predict the clinical course of the disease and affect a patient’s diagnosis and treatment plan. In a cross-sectional and longitudinal study involving 799 patients with a heterozygous mutation in the *GCK* gene in the UK, patients with a *GCK* gene mutation showed no significant changes in blood glucose levels and HbA1c levels after drug treatment (38). In this study, follow-up blood glucose monitoring after drug withdrawal of the proband showed that blood glucose control was sufficient and drug therapy was not required. In addition, several *GCK-MODY* patients in this family developed ocular complications (cataracts) or cardiovascular disease (hypertension, stroke) later in life. Interestingly, these complications are relatively mild and did not significantly increase their mortality.

In conclusion, for patients with a high clinical suspicion of MODY, a definite diagnosis should be made through genetic testing. Correct diagnosis helps patients to choose a personal treatment plan and avoid overtreatment. In our study, we reported a *GCK-MODY* family with a mutation in exon 9 of the *GCK* gene c.1160C > T (p.Ala387Val), which, according to our knowledge, was the first time to be detected in the Chinese population. For this *GCK-MODY* family, exercise and diet were sufficient as a therapeutic approach. The underlying molecular mechanism of action of *GCK* gene c.1160C > T (p.Ala387Val) participation in diabetes remains to be elucidated and future prospective investigations are warranted.

## Data Availability Statement

The datasets presented in this study can be found in online repositories. The names of the repository/repositories and accession number(s) can be found below: NCBI SRA: PRJNA744432.

## Ethics Statement

Written informed consent was obtained from the individual(s) for the publication of any potentially identifiable images or data included in this article.

## Author Contributions

Conceived and designed the experiments: JL and HZ. Performed the experiments: JL, XW, AD, CW, JW, SJ, and HZ. Analyzed the data: JL and MS. Wrote the paper: MS and JL. JL and MS contributed equally. All authors contributed to the article and approved the submitted version.

## Funding

This study was supported by Grants from the National Natural Science Foundation of China (81900719, 81800704), Grants from the Health and Family Planning Commission of Wuhan City (Grant Number WX18M02).

## Conflict of Interest

The authors declare that the research was conducted in the absence of any commercial or financial relationships that could be construed as a potential conflict of interest.

The handling editor declared a shared affiliation with the authors at time of review.

## Publisher’s Note

All claims expressed in this article are solely those of the authors and do not necessarily represent those of their affiliated organizations, or those of the publisher, the editors and the reviewers. Any product that may be evaluated in this article, or claim that may be made by its manufacturer, is not guaranteed or endorsed by the publisher.
